# Case Report: Simultaneously Developed Amyopathic Dermatomyositis and Autoimmune Sclerosing Cholangitis – a Coincidence or a Shared Immunopathogenesis?

**DOI:** 10.3389/fimmu.2022.825799

**Published:** 2022-02-23

**Authors:** Tomislav Ledenko, Iva Sorić Hosman, Marijana Ćorić, Alenka Gagro

**Affiliations:** ^1^ Department of Pediatrics, Zadar General Hospital, Zadar, Croatia; ^2^ Department of Pathology and Cytology, University Hospital Centre Zagreb, Zagreb, Croatia; ^3^ School of Medicine, University of Zagreb, Zagreb, Croatia; ^4^ Division of Pulmonology, Allergology, Immunology and Rheumatology, Department of Pediatrics, Children’s Hospital Zagreb, Zagreb, Croatia

**Keywords:** juvenile dermatomyositis, amyopathic dermatomyositis, autoimmune liver disease, autoimmune sclerosing cholangitis, shared autoimmunity, myositis specific antibodies

## Abstract

Inflammatory rheumatic diseases (IRD) and autoimmune liver diseases (AILD) share many similarities regarding epidemiology, genetics, immunology and therapeutic regimens, so it is not surprising that approximately 20% of patients with AILD are diagnosed with an IRD as well. Clinical features and biochemical hallmarks of IRD and AILD often intertwine and cross diagnostic criteria. Therefore, the real distinction of underlying disorders in a patient with these comorbidities may be challenging. The present report is the first report of simultaneously developed juvenile dermatomyositis (JDM) and autoimmune sclerosing cholangitis (ASC) with both entities fulfilling the latest guidelines for a definite diagnosis. Both of these diagnoses are difficult to definitely establish since ASC has a similar serologic profile as autoimmune hepatitis and liver histological analysis is frequently non-specific, whereas clinically amyopathic JDM diagnosis depends mostly on classical dermatological symptoms, while the rest of the diagnostic criteria, including the necessity for skin or muscle biopsy and the presence of myositis specific antibodies, are still not uniformed. In spite of these challenges, our patient clearly met European League Against Rheumatism/American College of Rheumatology classification criteria for CAJDM and The European Society for Pediatric Gastroenterology, Hepatology and Nutrition diagnostic criteria for ASC. Since elevated serum transaminases, the presence of serum antinuclear antibodies and hypergammaglobulinemia could be explained as a part of both JDM and ASC, the underlying pathophysiology remains debatable. Intriguingly, JDM and ASC share genetic predisposition including human leukocyte antigen allele DRB1*0301 and tumor necrosis factor α 308A allele. Furthermore, both humoral and cellular components of the adaptive immune system contribute to the pathogenesis of JDM and ASC. Moreover, recent findings indicate that the loss of the CD28 expression on T-cells plays a significant role in their pathogenesis along with the Th17 immune pathway. Despite these common features that suggest shared autoimmunity, AILD and autoimmune myositis are traditionally studied and managed independently. The lack of therapies that target the underlying cause results in a high rate of adverse events due to unspecific immunosuppressive therapy. Shared autoimmunity is an ideal area to develop new, targeted immunotherapy that would hopefully be beneficial for more than one disease.

## Introduction

Juvenile dermatomyositis (JDM) is an autoimmune inflammatory disease affecting skeletal muscle tissue and skin, with an incidence of 3.2 per million children per year (1, 2). Clinically amyopathic JDM (CAJDM), or more recently called skin-predominant JDM, is DM subphenotype which includes hallmark cutaneous manifestations of DM without muscle weakness lasting for at least 6 months, affecting only around 10% of all JDM patients (3, 4). Autoimmune sclerosing cholangitis (ASC) is autoimmune liver disease (AILD) affecting intrahepatic and extrahepatic bile ducts, with an incidence of 1 per million children per year (5). The diagnosis is based on elevated liver enzymes, serological profile similar to autoimmune hepatitis (AIH) and bile duct lesions visualized on cholangiography (6). Therefore, a concomitant development of both of these autoimmune diseases in the same patient is exceptional.

Among patients with autoimmune myositis (AIM), elevated serum liver enzymes are a common phenomenon. Patients with DM frequently have simultaneously elevated creatine kinase (CK), aspartate aminotransferase (AST) and alanine aminotransferase (ALT) at the presentation, usually of skeletal muscle origin. Serum transaminases usually normalize proportionally to CK serum level (7). Consequently, concomitantly elevated CK, AST and ALT may be attributed to myositis leaving a concurrent liver disease unrecognized, while elevated transaminases without a determination of serum CK level may be misdiagnosed as a liver disease.

Along with elevated liver enzymes, patients with AIM as well as those with AILD often have positive antinuclear antibodies (ANA) and hypergammaglobulinemia. Therefore, in a case of increased transaminases without elevation of serum CK in patients with myositis, additional liver disease should be excluded. Here we report an extraordinary case of JDM and ASC co-occurrence and discuss shared clinical, biochemical, immunological and genetic features of these two distinct clinical entities.

## Case Report

**Figure 1:**
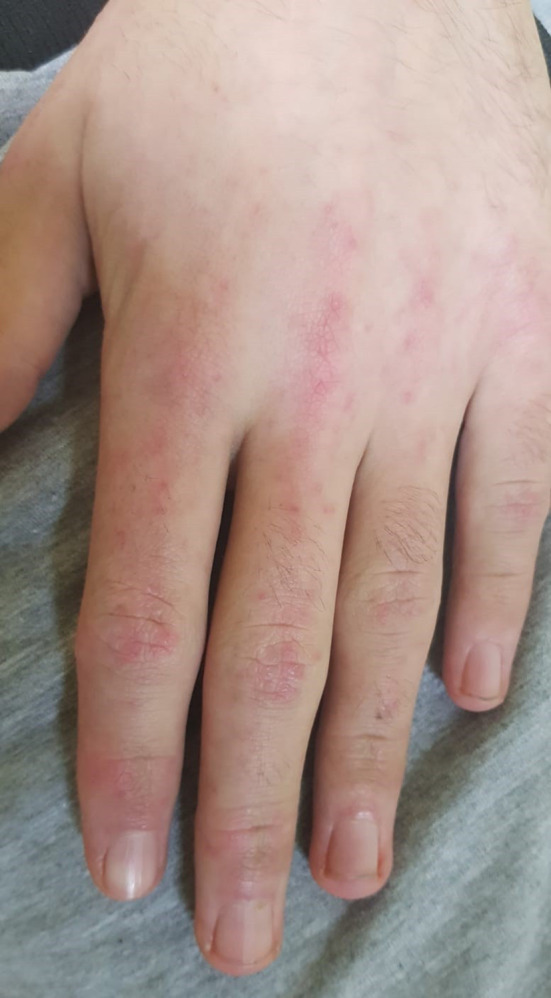
Characteristic cutaneous features of DM called Gottron’s papules (multiple erythematous papules over the dorsal aspect of metacarpophalangeal and interphalangeal joints).

**Figure 2:**
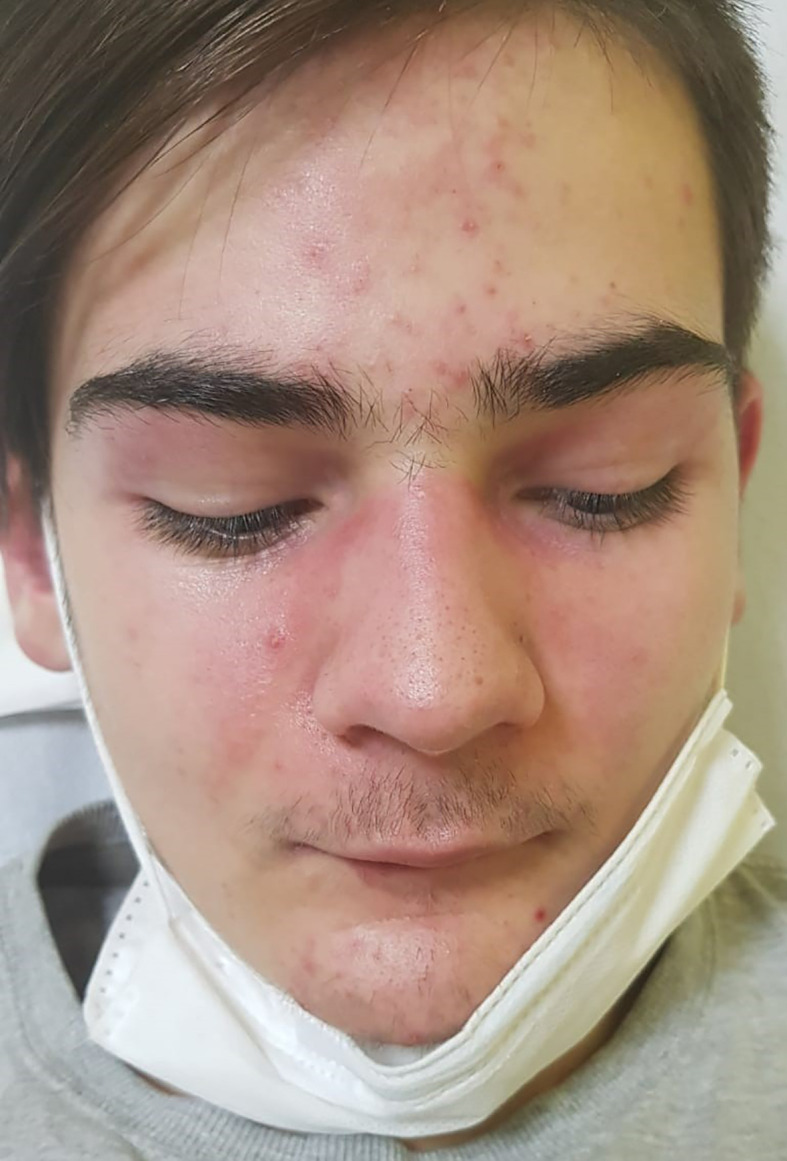
Characteristic cutaneous features of DM called heliotrope rash (violaceous erythema of the eyelids or periorbital skin) and the malar rash.

A 16-years old, previously healthy boy presented at our Department of Pediatrics with a 12-months history of skin rash. There was no history of photosensitivity, oral ulcers, alopecia, Raynaud’s phenomenon, joint pain, morning stiffness, fever, myalgia or weight loss. Physical examination revealed characteristic cutaneous features of DM including Gottron’s papules (multiple erythematous papules over the dorsal aspect of metacarpophalangeal and interphalangeal joints, **Figure 1**), Gottron’s sign (violaceus macules over extensor surfaces of elbows and medial malleoli), heliotrope rash (violaceous erythema of the eyelids or periorbital skin) and the malar rash, including the nose bridge (**Figure 2**). The rest of the physical examination was unremarkable. There was no clinically detectable muscle weakness. Chest radiograph, echocardiogram and renal ultrasound were normal.

Laboratory investigations were: C-reactive protein 4.2 mg/l (normal <3 mg/l), erythrocyte sedimentation rate 11 mm/h (normal <12 mm/h), AST 69 U/l (normal 10–42 U/l), ALT 107 U/l (normal 10–37 U/l), γ-glutamyltransferase 178 U/l (normal 10-43 U/l). Serum CK levels and lactate dehydrogenase were within normal range (233 U/l and 287 U/l, respectively). Fecal calprotectin was 316 mcg/g and fecal occult blood test was negative. Immunoglobulin (Ig) G levels were increased up to 22.1 g/l (normal 7-16 g/l) as well as total serum protein and albumin to globulin ratio. The rest of the laboratory findings where within the reference range, including blood count, coagulogram, urea, creatinine, alkaline phosphatase, bilirubin, complement (C3, C4), anti-tissue transglutaminase antibodies, rheumatoid factor, anti- cyclic citrullinated peptide antibodies, alpha1-antitrypsin, copper, ceruloplasmin and urine analysis (including proteinuria and copperuria). Hepatitis A, B, C, EBV, CMV, Toxoplasma and HSV1 serology as well as quantiferone test were negative.

Immunological blood tests revealed positive ANA, while antibodies against double-strained DNA and extractable nuclear antigens were negative. Furthermore, cytoplasmic (c-) and atypical perinuclear (a p-) anti-neutrophil cytoplasmic antibodies (ANCA) were positive, whereas p-ANCA were firstly negative, but on a second examination were positive. Anti-cardiolipin (ACA), anti-beta2 glycoprotein I (B2GPI) and anti- glycoprotein 210 (gp210) antibodies were also positive. Anti-liver/kidney microsomal type 1(LKM-1), anti-liver cytosol type 1 (LC-1), anti-smooth muscle antibodies (SMA), anti-soluble liver antigen (SLA) and anti-mitochondral (AMA) antibodies were all negative. Amongst myositis specific antibodies (MSA) only anti-transcriptional intermediary factor 1 gamma (anti-TIF1γ) antibodies were positive.

Although serum CK levels were within normal range, electromyography showed occasional polyphasy and decreased action potential amplitudes on both peroneal nerves. Furthermore, our patient was positive for myositis specific anti-TIF1γ antibodies, which are, according to the most recent guidelines, an additional criterion for diagnosing skin-predominant DM (8, 9). Based on the European League Against Rheumatism/American College of Rheumatology (EULAR/ACR) classification criteria for idiopathic inflammatory myopathies and more recently proposed classification criteria by the Delphi project group our patient was diagnosed with CAJDM (**Tables 1**, **2**) (2, 8).

**Table 1:** Clinical practice guidance table for the diagnosis of juvenile dermatomyositis (9) and results in our patient.

EXAMINATION	FINDING	FINDINGS IN OUR PATIENT
1) Dermatological symptom	a) Heliotrope rash: reddish purple edematous erythema in the eyelids, unilateral or bilateral	a) +
	b) Gottron’s signs: Erythematous or violaceous macules on extensor surface of the finger joints with hyperkeratosis and dermatrophy	b) +
	c) Erythema of extensor surfaces of the joints of the elbow, knee etc.*^1^.	c) +
	d) Findings of skin biopsy consistent with dermatomyositis^*2^	d) ND
2) Muscular symptom	Muscle weakness of proximal muscles in upper or lower extremities^*3^	–
3) Imaging	Findings indicating myositis with MRI: High intensity on T2 weighted/fat suppression MRI and normal intensity on T1 weighted MRI	ND
4) Biochemical examination	Elevated serum level of muscle enzymes (creatine kinase or aldolase)	–
5) Immunological examination	Positive result for myositis-specific autoantibodies^*4^	+
6) Pathological examination	Pathological findings indicating myositis with muscle biopsy (degeneration of muscle fibers and cellular infiltration)	ND
7) Exclusions	Myositis caused by infections, eosinophilic myositis, autoinflammatory diseases with rash similar to DM such as Nakajo-Nishimura syndrome, drug-induced myopathy, myopathy due to endocrine abnormality or congenital anomaly, muscle symptoms due to electrolyte abnormality, muscular dystrophy and other congenital muscular diseases, muscle weakness due to central or peripheral neuropathy, psoriasis, eczema, and other related diseases with other causes such as allergy.	–
**DIAGNOSIS**	**Classical JDM**: The presence of 1 or more items of (1) dermatological symptoms (a) to (c) (2), muscular symptom and 2 or more items of (3) to (6) during the follow-up without any of items of (7).	**-**
	**JHDM**: The presence of 1 or more items of classical dermatological symptoms (a) to (c), and 1 or more items of findings indicating myositis (3) to (6) without clinical muscle weakness without any of items of (7).	**+**
	**JADM**: The presence of 1 or more items of classical dermatological symptoms (a) to (c) without any evidences of myositis (2) to (6) without any of items of (7).	**-**
	**JPM**: The presence of 3 or more items of (2) to (6) without dermatological symptoms (1) without any of items of (7).	**-**

*^1^Ulcerative lesions or secondary infection may modify the appearance of rash.

*^2^Hyperkeratosis, vacuolation of basal keratinocytes, deposition of melanin, perivascular lymphocyte infiltration, increased dermal edema, mucin deposition and thickening or atrophy of skin are observed, but it is difficult to differentiate JDM from SLE by pathological findings alone. Thus, single symptom is not adopted as a dermatological finding.

*^3^Muscle weakness ranges from mild ones (e.g., stumbling, new onset of difficulties in exercise) to advanced ones (e.g., difficulties in standing up from sitting position or rolling over in the bed).

*^4^Although only anti-Jo-1 antibodies could be measured at the time of revision of these guidelines, anti-ARS, anti-MDA5, anti-Mi-2, and anti-TIFI-ɣ antibodies are currently listed on the national health insurance price list.

^+^, Positive finding; -, Negative finding; ND, not done; JDM, juvenile dermatomyositis; JHDM, juvenile hypomyopathic dermatomyositis; JADM, juvenile amyopathic dermatomyositis; JPM, juvenile polymyositis.

**Table 2:** EULAR/ACR classification criteria table for adult and juvenile idiopathic inflammatory myopathies and their major subgroups (2) and score in our patient.

Variable	Finding	Without muscle biopsy	With muscle biopsy	Score in our patient
Age of onset	Age of onset of first symptom assumed to be related to the disease ≥ 18 years and < 40 years	1.3	1.5	0
	Age of onset of first symptom assumed to be related to the disease ≥ 40 years	2.1	2.2	0
Muscle weakness	Objective symmetric weakness, usually progressive, of the proximal upper extremities	0.7	0.7	0
	Objective symmetric weakness, usually progressive, of the proximal lower extremities	0.8	0.5	0
	Neck flexors are relatively weaker than neck extensors	1.9	1.6	0
	In the legs proximal muscles are relatively weaker than distal muscles	0.9	1.2	0
Skin manifestations	Heliotrope rash: Purple, lilac-colored or erythematous patches over the eyelids or in a periorbital distribution, often associated with periorbital edema	3.1	3.2	3.1
	Gottron´s papules: Erythematous to violaceous papules over the extensor surfaces of joints, which are sometimes scaly. May occur over the finger joints, elbows, knees, malleoli and toes	2.1	2.7	2.1
	Gottron’s sign: Erythematous to violaceous macules over the extensor surfaces of joints, which are not palpable	3.3	3.7	3.3
Other clinical manifestations	Dysphagia or esophageal dysmotility	0.7	0.6	0
Laboratory measurements	Anti-Jo-1 (anti-histidyl-tRNA synthetase) autoantibody present	3.9	3.8	0
	Elevated serum levels of creatine kinase (CK) or lactate dehydrogenase (LDH) or aspartate aminotransferase (AST) or alanine aminotransferase (ALT)	1.3	1.4	1.3
Muscle biopsy features- presence of:	Endomysial infiltration of mononuclear cells surrounding, but not invading, myofibres		1.7	0
	Perimysial and/or perivascular infiltration of mononuclear cells		1.2	0
	Perifascicular atrophy		1.9	0
	Rimmed vacuoles		3.1	0
**TOTAL SCORE**	Probability of IIM without muscle biopsy=1/[1+exponential(5.33–score)] or probability of IIM including muscle biopsy=1/[1+exponential(6.49–score)] or by using a web calculator at www.imm.ki.se/biostatistics/calculators/iim			**9.8 ➔ 99% probability of IIM diagnosis (JDM subgroup)**

IIM, idiopathic inflammatory myositis; JDM, juvenile dermatomyositis.

**Figure 3:**
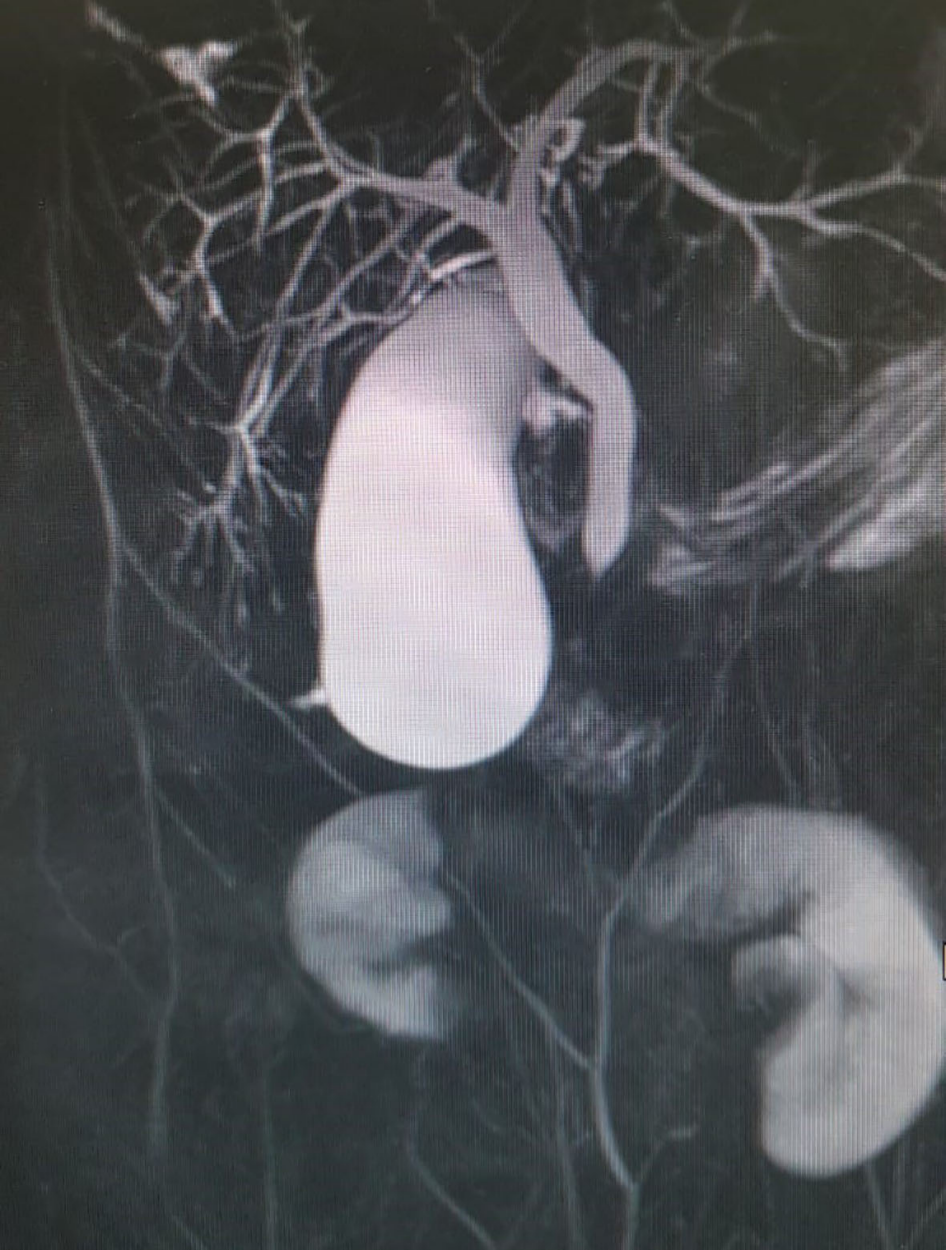
Distended common bile duct and intrahepatic bile ducts **on** magnetic resonance cholangiopancreatography (MRCP).

**Figure 4:**
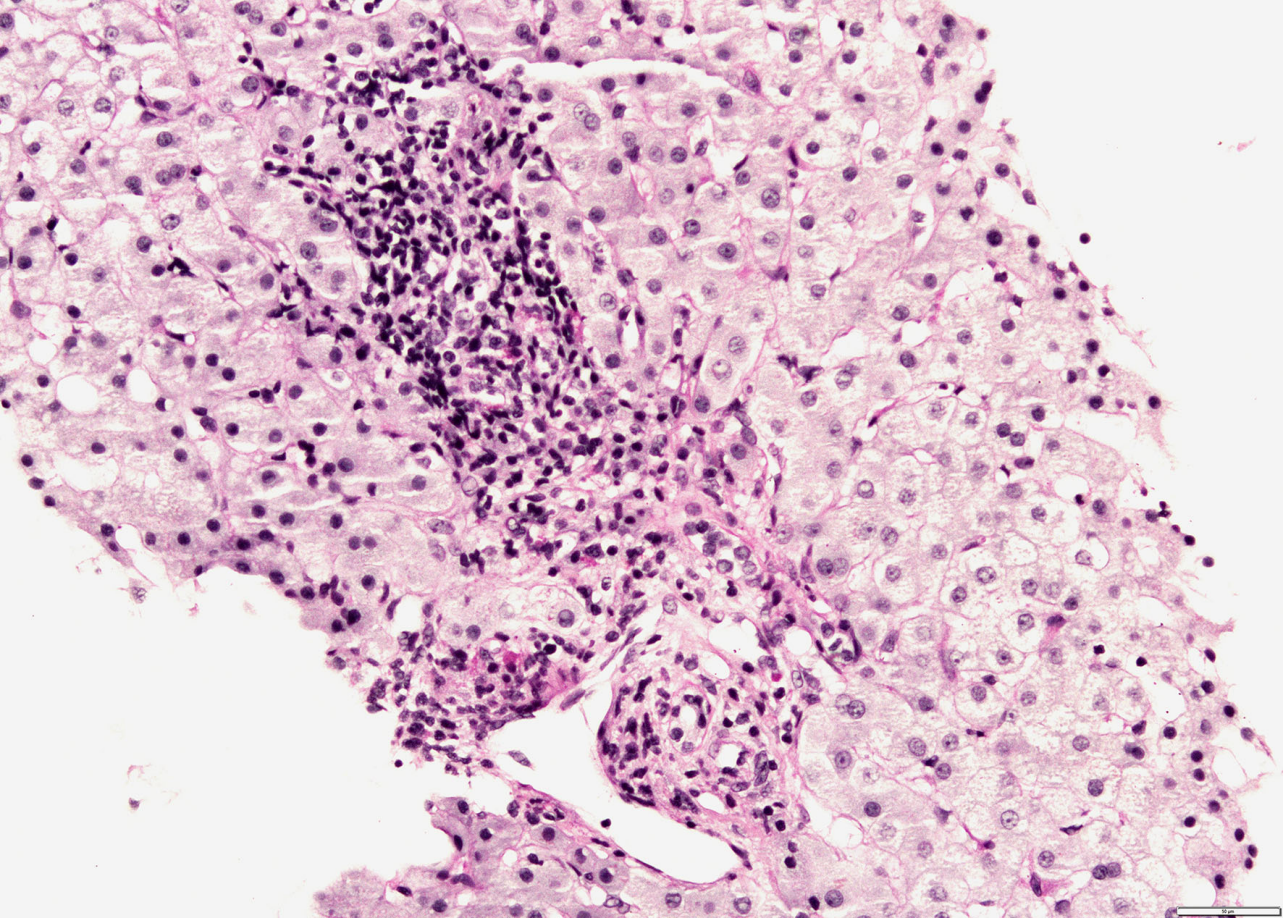
Liver biopsy shows mononuclear infiltration of portal tract with focal interface hepatitis (PAS-D stain, magnification 200x).

Since there was no correlation between serum transaminases and CK levels, other source of transaminases, rather than skeletal muscles, was suspected. Abdominal ultrasound revealed dilated common bile duct and magnetic resonance cholangiopancreatography (MRCP) confirmed distended common and intrahepatic bile ducts (**Figure 3**). Finally, histological analysis of the liver biopsy specimen showed a dense mononuclear cell infiltration with peripheral piecemeal necrosis in 1 out of the total 10 portal places encompassed within the specimen (**Figure 4**). Although the histological finding was non-specific, it was compatible with the diagnosis of ASC. Based on the European Society for Pediatric Gastroenterology Hepatology and Nutrition (ESPGHAN) scoring system ASC diagnosis was finally established (**Table 3**) (6).

**Table 3:** ESPGHAN table of scoring criteria for the diagnosis of juvenile autoimmune liver disease (6) and results in our patient.

VARIABLE	CUT-OFF	POINTS		OUR PATIENT
		AIH	ASC	
ANA	≥ 1:20^#^	1	1	+
and/or SMA*	≥ 1:80	2	2	–
Anti-LKM-1* or	≥ 1:10^#^	1	1	–
	≥ 1:80	2	1	–
Anti-LC-1	Positive^#^	2	1	–
Anti-SLA	Positive^#^	2	2	–
pANNA	Positive	1	2	+
IgG	> ULN	1	1	–
	> 1.20 ULN	2	2	+
Liver histology	Compatible with AIH	1	1	+
	Typical of AIH	2	2	–
Absence of viral hepatitis (A,B,E,EBV), NASH, Wilson disease & drug exposure	Yes	2	2	+
Presence of extrahepatic autoimmunity	Yes	1	1	+
Family history of autoimmune disease	Yes	1	1	–
Cholangiography	Normal	2	-2	–
	Abnormal	-2	2	+
**TOTAL POINTS**		≥ 7: probable AIH;	≥ 7: probable ASC;	6 for AIH
		≥ 8: definite AIH	≥ 8: definite ASC	11 for ASC

AIH, autoimmune hepatitis; ASC, autoimmune sclerosing cholangitis; ANA, anti-nuclear antibody; SMA, anti-smooth muscle antibody; anti-LKM-1, anti-liver kidney microsomal antibody type 1; anti-LC-1, anti-liver cytosol type 1; anti-SLA, anti-soluble liver antigen; IgG, immunoglobulin G; EBV, Epstein Barr virus; NASH, non-alcoholic steatohepatitis; ULN, upper limit of normal.

*Antibodies measured by indirect immunofluorescence on a composite rodent substrate (kidney, liver, stomach).

^#^Addition of points achieved for ANA, SMA, anti-LKM-1, anti-LC-1 and anti-SLA autoantibodies cannot exceed a maximum of 2 points.

After being diagnosed with both CAJDM and ASC, our patient was managed in an interdisciplinary manner. Since high-dose steroid is the preferred initial treatment for both JDM and ASC, the patient commenced prednisone at a dose of 60 mg/day. The dose was gradually decreased in parallel to the decline of serum transaminases. Azathioprine (100 mg/day) was added as a steroid-sparing agent (6, 9). According to the ESPGHAN protocol, ursodeoxycholic acid (UDCA, 750 mg/day) was also administered (6). Because of the deterioration of skin symptoms despite a good patient’s compliance, 6 months after the therapy initiation, hydroxychloroquine (200 mg/day), which is recommended for skin predominant JDM by the Children’s Arthritis and Rheumatology Research Alliance (CARRA), was added to the therapy regimen (10). Eventually, the treatment resulted in clinical and laboratory improvement. Since the patient’s follow-up to this date is only a year long, further interdisciplinary monitoring is planned.

With an aggressive early treatment, 30–50% of patients have the potential to reach remission within 2–3 years of disease onset with a mortality rate of less than 4%, whereas 40-60% of patients have a persistently active disease leading to complications like calcinosis, persistent muscle weakness, skin or muscle atrophy and lipodystrophy (11). Based on Single Hub and Access point for pediatric Rheumatology in Europe (SHARE) recommendations, biologics that include intravenous immunoglobulin (IVIG) in immunomodulatory dosage as well as B cell depletion therapy (rituximab) may be a useful adjunct for resistant or refractory disease. IVIG are given to patients with prominent skin features and it usually takes 2-3 months of therapy for the clinical improvement. The effect of rituximab can take up to 26 weeks to be seen. Anti-TNF agents may also be considered in a refractory disease; infliximab or adalimumab are favored over etanercept. The SHARE group recommends withdrawing treatment if a patient has been off steroids and in remission on a disease-modifying antirheumatic drug a minimum of 1 year (11).

## Discussion

### Classification and Diagnosis

Skin-predominant DM or CADM can be challenging to diagnose since, until recently, it has not been recognized by the most of classification guidelines in the field. As a consequence, more than a half of these patients have had an alternate diagnosis prior to eventually being diagnosed with DM, with a median diagnostic delay of 15 months. The most common alternate diagnosis was systemic lupus erythematosus (SLE) (12). This is not surprising considering skin lesions occur very early in the disease course in both SLE and DM and show a similar distribution. In order to resolve the existing classification dilemma for patients with CADM, the latest EULAR/ACR classification criteria for inflammatory myopathies include three DM associated skin manifestations: Gottron’s sign, Gottron’s papules and heliotrope rash (2). In spite of this enormous improvement, approximately 26% of patients with CADM still do not meet criteria for a definite classification (13). For this reason, additional efforts have been made to develop skin-focused classification criteria. With the identification of MSA, new phenotypes have been characterized depending on the type of cutaneous lesions, autoantibody profile, systemic involvement and outcome (14). The presence of MSA has been proposed as an additional criterion when classifying patients with CADM (8, 9).

Our patient was diagnosed with CAJDM based on hallmark cutaneous manifestations of DM (Gottron’s papules, Gottron’s sign, heliotrope rash) lasting for more than 6 months with no clinical signs of myositis. Since he had electromyographic signs of muscle involvement and positive MSA, skin biopsy was not performed, even though EULAR/ACR classification guidelines recommend skin biopsy in patients without muscle involvement. However, the EULAR/ACR criteria do not include MSA except for anti-Jo-1 autoantibody, whereas the latest guidelines include positive anti-TIF1γ autoantibodies as an additional criterion (8, 9). CAJDM includes two subphenotypes named amyopathic JDM and hypomyopathic JDM, with the latter group having evidence of subclinical myositis upon laboratory, electrophysiological and/or radiologic evaluation (15). Accordingly, our patient appertains to the hypomyopathic group. Analysis of hypomyopathic DM patients in the literature shows that none of the reported adult or juvenile hypomyopathic DM patients, despite of laboratory (abnormal CK), electrophysiological (abnormal EMG) and/or radiologic (MRI) evidence of myositis, developed clinically significant myositis during a mean follow-up exceeding 5 years (15–18). Therefore, the presence of subclinical myositis is not predictive of a future clinically significant myositis.

Elevated transaminases are frequently found in patients with AIM. This elevation generally follows the trend of serum CK level suggesting the skeletal muscle, rather than the liver, as the source of transaminases. Approximately 80% of all patients with AIM have elevated serum transaminases at the time of presentation and their serum level normalization following the normalization of CK levels, confirming the strong correlation between CK level and transaminases (7, 19). Therefore, evaluation for additional liver disease is not routinely required. However, if elevated liver enzymes do not follow serum CK level, further evaluation is required. A wide range of hepatic disorders in patients with AIM have been reported, including fatty liver, hepatic congestion, non-specific reactive hepatitis and AILD (20). Mostly reported concomitant rheumatic diseases in patients with AILD are SLE, rheumatoid arthritis, Sjogren’s syndrome and systemic sclerosis, while concurrent AIM is extremely rarely observed. Only a few cases of AIH type 1 patients with polymyositis have been reported to this date (21–27) and only one case each AIH type 1 (28) and AIH-primary biliary cirrhosis overlap syndrome (29) associated with DM. However, all of the reported patients were adults and had at least one additional autoimmune disease. To our knowledge, the present case is the first report of concomitant DM and ASC (or even AILD in general) development in pediatric population.

ASC affects about a half of children presenting with AILD. These patients usually have the same serological profile and liver histology as AIH but with an abnormal cholangiography finding already at presentation (30, 31). Since approximately 25% of the children with ASC have no histological features suggesting bile duct involvement, cholangiography is crucial for diagnosis. However, International Autoimmune Hepatitis Group (IAIHG) scoring systems do not include cholangiography at the disease onset (32). A prospective study showed that almost a half of the children whit serological and histological features of AIH have bile duct damage demonstrable by cholangiography (30). Therefore, although cholangiography in adults is recommended in a case of cholestasis, it is mandatory in children with AIH or ASC regardless of cholestatic enzyme levels and abnormal cholangiography findings have been proposed as an additional criterion when diagnosing ASC (6). Although in our patient the liver histology was not typical of AIH, it was compatible with AIH and MRCP was abnormal. Additional fulfilled ESPGHAN criteria for ASC diagnosis in our patient were positive ANA, apANCA, high titer IgG, presence of extrahepatic autoimmunity and the absence of viral hepatitis, non-alcoholic steatohepatitis, Wilson’s disease and drug exposure (6).

### Autoantibodies

ANA positivity is common in a number of autoimmune liver and rheumatic diseases, as well as in up to 15% of healthy individuals (33). MSA, on the other hand, are highly specific antibodies for AIM and are not identifiable in other rheumatic diseases (34). Our patient was positive with anti-TIF1γ, the most common MSA identifiable in 20 -35% of JDM patients (34, 35). Patients with CADM more frequently have anti-TIF1γ autoantibodies than DM patients (75 vs. 37%) (36). This autoantibody is associated with more severe cutaneous lesions, rash in photo-exposed pattern, a chronic disease course, focal or generalized lipodystrophy and milder muscle involvement (35, 37–40). Raynaud’s phenomenon and arthritis, as well as interstitial lung disease, are less frequent in anti-TIF1γ positive patients, but adults seem to have a much higher risk (>5x) for an associated internal malignancy (paraneoplastic DM), whereas anti-TIF1γ positive juvenile patients have a decreased association with internal malignancy (11, 41).

Antiphospholipid antibodies, including anti-cardiolipin (ACA) and anti-beta 2 glycoprotein I (anti-B2GPI) antibodies, exhibit a broad range of target specificities and affinities and can be positive in a wide range of autoimmune diseases. Though they have not been associated with DM, anti-B2GPI antibodies are frequently positive in patients with AILD and are associated with a large bile-duct involvement, increased mortality and increased cholangiocarcinoma risk in these patients (42). Interestingly, our patient was also positive with anti-gp210 antibodies, autoantibodies against the nuclear pore complex protein, which are highly specific of primary biliary cholangis (PBC), but have not been associated with other liver (autoimmune or non-autoimmune) or non-liver autoimmune diseases (43). In patients with PBC, positive anti-gp210 antibodies are an independent predictor of poor prognosis, a more rapid disease progression and a lower 5-year transplant-free survival compared to anti-gp210 negative patients. Furthermore, the degree of anti-gp210 expression is positively correlated with the intensity of inflammation around small bile ducts. The persistence of anti-gp210 after UDCA treatment is a risk factor for the progression to end-stage hepatic failure, whereas the disappearance of anti-gp210 after therapy indicates a more favorable clinical course in PBC (43). However, a possibility of a prognostic role of positive anti-gp210 antibodies in patients with ASC remains to be investigated.

Perinuclear ANCA (pANCA), though commonly associated with ASC, were initially negative in our patient, but on a second examination were positive, possibly due to the fact that the presence of ANA may interfere with pANCA detection, since neutrophil nuclei are stained by ANA, masking the perinuclear staining by pANCA (44). Atypical pANCA (or perinuclear anti-neutrophil nuclear antibodies, pANNA) have been associated with both ASC and AIH with a higher prevalence in patients with ASC (30, 45) and are included in both IAIHG and ESPGHAN criteria for AILD diagnosis. Even though the clinical significance of ANCA in these disorders remains unclear, detection of pANCA in a pediatric patient with hepatitis of unknown etiology points toward the diagnosis of AIH or ASC with the cholangiography finding crucial for the distinction between these two clinical entities.

### Autoimmune Mechanisms and Genetic Predisposition

In both ASC and JDM, along with humoral immunity, features of cellular immunity also play a prominent role in the disease development. Autoantibodies may be the actual pathogenic agents or they may occur secondary as a consequence of tissue damage by autoreactive T cells.

Defective regulatory T cells (CD4^+^CD25^+^ T cells) play a major role in the loss of tolerance that leads to autoimmune disease since these cells control the process of autoantigen recognition by preventing proliferation of autoreactive T cells. In children with AILD, regulatory T cells are defective in number and function compared with healthy controls and even when compared between patients at disease onset and those in remission (46). Regulatory T cells in children with AILD produce interferon (IFN) -γ and interleukin (IL)-17 and thereby sustain inflammation and hepatic damage. This is reflected in peripheral blood and liver by increased number of Th17 cells, IFN-γ and IL-17 (47). Intriguingly, an increased expression of these cells and cytokines was also found in serum, muscle and skin tissue of patients with DM (48, 49). In addition to that, one study found even higher gene expression of Th17 related cytokines in JDM than DM patients, indicating that Th17 pathway plays a more prominent role in the pathogenesis of JDM than DM (50).

Furthermore, these two distinct clinical entities share genetic predisposition. Both ASC and JDM are associated with higher frequency of HLA-DRB1*0301 allele (51–53). In HLA-DRB1*0301-positive individuals, TNF-α and IFN-γ secretion is increased (54). Furthermore, both JDM and ASC patients have a higher frequency of the TNFα-308A allele than healthy controls and this genotype is associated with a higher serum and local (muscle fibres and liver, respectively) expression of TNFα (55, 56). TNF-α induces CD28 loss in T cells (57). The loss of CD28 expression on CD8^+^ T-cells, or so-called aging of T-cells, appears to play an important role in the pathogenesis of both JDM and ASC. CD8^+^CD28^-^ T cells are apoptosis-resistant and are easily triggered to produce proinflammatory cytokines, mostly IFN-γ and TNF-α (57). In JDM patients, a high frequency of these cells is present in circulation and muscle tissue (58), whereas in AILD patients these cells accumulate in liver and localize around bile ducts (57). CD28^-^ T cells can increase adhesion molecule expression on biliary epithelial cells and, consequently, contribute to the activation of cytolitic mechanisms leading to cholangiocyte apoptosis (57).

On top of all that, disease onset in both ASC and JDM has been associated with infective agents as a trigger. Most of the JDM patients (>60%) report symptoms of a respiratory or gastrointestinal infection around 3 months before the disease onset (59), while in ASC pathogenesis a possible role of bacteria has been proposed, since atypical pANCA cross-react with the bacterial cell division protein (60). In addition to that, a molecular mimicry has been described with protein products of the Escherichia coli mutY gene and Salmonella typhimurium mutB gene and the gp210 epitope, suggesting that anti-gp210 antibodies may arise by molecular mimicry of bacterial antigenic determinants (61). However, since defective regulatory T cells, increased IL-17 production, HLA-DRB1*0301 allele, TNFα-308A allele and an infective trigger are a common findings in a wide range of autoimmune disease, these shared autoimmune mechanisms and genetic predisposition do not necessary mean a shared autoimmunity.

Considering the important role TNF-α clearly has in the pathogenesis of both ASC and JDM, TNF-α inhibitors might be effective in both of these diseases as well as in a case of their co-existence. Indeed, effectiveness of infliximab has been reported in both refractory AILD and JDM (62–64). However, a number of reports on anti-TNF-α-induced AIH and AIM has been published (65–68), highlighting the dual effects that anti-TNF-α agents might have. This paradox of anti-TNF-α therapy has been mainly attributed to the disruption of the regulatory role of TNF-α signaling in immune pathways (68). In addition to that, the high risk for serious infections is a well-recognized side effect of anti-TNF-α therapy (63, 68), making TNF-α antagonists a therapeutic option only for refractory cases of AIH. A better understanding of the TNF-α role in the pathogenesis of these diseases is a prerequisite to introducing TNF-α-blockers to therapy. Furthermore, considering both of these diseases show defective regulatory T cells, introducing sirolimus (a therapeutic agent that selectively expands regulatory T cells) could be beneficial for more than one disease (69). Further research is required to elucidate benefits of biologic therapy for ASC and JDM.

## Conclusion

ASC and CAJDM are rare diseases that are challenging to diagnose and treat separately, but even more of a diagnostic and therapeutic challenge when coexisting. Since scoring systems for classification include interfering features like liver enzyme abnormalities, hypergammaglobulinemia and abnormal titer of serum ANA, the question of concomitant development of these two clinical entities in the same patient remains debatable. In addition to the mentioned interfering data, histological examination of a liver biopsy specimen in ASC and skin biopsy specimen in JDM often may not be helpful for a correct diagnosis due to a lack of specificity.

The present report is, to the best of our knowledge, the first report of simultaneously developed JDM and ASC with both entities fulfilling the latest guidelines for a definite diagnosis. Intriguingly, although extremely rarely associated, these two distinct clinical entities share multiple immunopathogenic features as well as genetic susceptibility factors. The present report emphasizes the need for further research in order to better understand underlying pathogenesis and resolve the remaining dilemmas in the field of shared autoimmunity. Furthermore, in inflammatory rheumatic diseases affecting the skin, including JDM, a more detailed characterization of skin lesions, serological markers and their correlation with disease outcomes and therapeutic responses is an imperative for a more individualized approach.

The coexistence of autoimmune liver and rheumatic diseases represents an ideal area for investigating and developing new therapeutic agents and personalized treatments. Research goals for the future should include further elucidation of shared autoimmune pathways across the spectrum of autoimmune diseases what will eventually lead to the identification of novel therapeutic targets. Targeted immunotherapy aimed at shared components of immunopathogenesis in frequently associated autoimmune diseases would be highly beneficial for more than one disease and would, consequently, minimize potential adverse effects of treating each comorbidity separately.

## Data Availability Statement

The original contributions presented in the study are included in the article/supplementary material. Further inquiries can be directed to the corresponding author.

## Ethics Statement

Written informed consent was obtained from the individual(s), and minor(s)’ legal guardian/next of kin, for the publication of any potentially identifiable images or data included in this article.

## Author Contributions

TL conceived the study and defined the concept. TL and AG cared for the patient and extracted the data from the hospital. MĆ performed the histological analysis. TL and IS wrote the initial draft of the manuscript. AG and MĆ reviewed the manuscript and contributed to the final draft. All authors contributed to the article and approved the submitted version.

## Funding

Open access publication fees for this article were provided by G-M Pharma Zagreb d.o.o. The funder had no role in the study design, collection, analysis, interpretation of data, the writing of this article or the decision to submit it for publication.

## Conflict of Interest

The authors declare that the research was conducted in the absence of any commercial or financial relationships that could be construed as a potential conflict of interest.

## Publisher’s Note

All claims expressed in this article are solely those of the authors and do not necessarily represent those of their affiliated organizations, or those of the publisher, the editors and the reviewers. Any product that may be evaluated in this article, or claim that may be made by its manufacturer, is not guaranteed or endorsed by the publisher.
